# Utilizing Evoked Response Potentials to predict clinical outcomes to Methylphenidate in children with Attention Deficit Hyperactivity Disorder

**DOI:** 10.1192/j.eurpsy.2026.10566

**Published:** 2026-07-31

**Authors:** M. Mathew, B. N. Patra, R. Sagar, R. Verma, R. Bhargava, S. Kaur

**Affiliations:** 1 Psychiatry; 2 Physiology, All India Institute of Medical Sciences, New Delhi, India

## Abstract

**Introduction:**

Attention Deficit/ Hyperactivity disorder is a neurodevelopmental disorder characterised by persistent pattern of hyperactivity, impaired attention and poor organization. ADHD is associated with multiple neurophysiological deficits leading to impairment in sustaining attention, reward processing and regulation of behavior. There is marked intra- individual variation in presentation due to the diagnostic, developmental and neurobiological heterogeneity which can make treatment challenging. Identifying biological predictors of treatment response becomes imperative to develop personalized regimens for the various subsets. Evoked response potentials (ERP) allow the assessment of sensory and cognitive processes and can be potentially useful.

**Objectives:**

This study endeavors to explore the potential Evoked response potentials in predicting medication response.

**Methods:**

Longitudinal observational study, with sample size of 30 male cases of ADHD aged between 8-16 years who were planned to be started on Methylphenidate by their treating psychiatrist. Informed consent from parents and assent from the study participants were taken. Severity of ADHD was assessed using Conner’s parent rating scale (CPRS). Prior to starting treatment, a 32 channel electroencephalograph (EEG) recording was taken while the participants were performing an auditory oddball task. Amplitude and latencies of P300 and N200 (evoked potentials) were extracted from the EEG data for 3 central channels (Cz, Oz, Pz) . Participants were divided into good and poor responders following 4 weeks of treatment and baseline ERP data was compared between both groups.

**Results:**

P300 amplitude and latency across Auditory Oddball task did not reveal significant differences between Good and Poor Responders except P300 latency of the target stimuli over Cz channel location, participants who responded better to medication had significantly lesser latency as compared to the other group. P300 amplitude at Cz and Pz of non target stimuli correlated negatively with improvement in hyperactivity and total symptom score on CPRS- lower P300 amplitude predicted better improvement (p=0.02, 0.04 at Cz and Pz respectively).

N200 of non target stimuli had a significantly smaller amplitude in good responders as compared to poor responders in our study. A significant correlation was found between the amplitude and improvement in inattention score (rho 0.45, p-0.01) on Cz location. N200 latency was also found to be significantly longer in good responders and correlated with improvement in CPRS scores at all three channel locations.

**Image 1:**
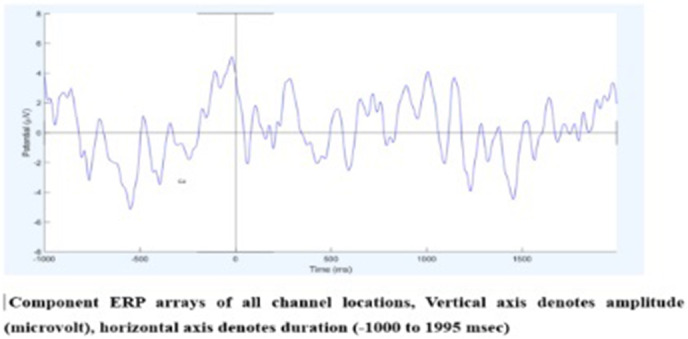
Image 1: Long description.

**Image 2:**
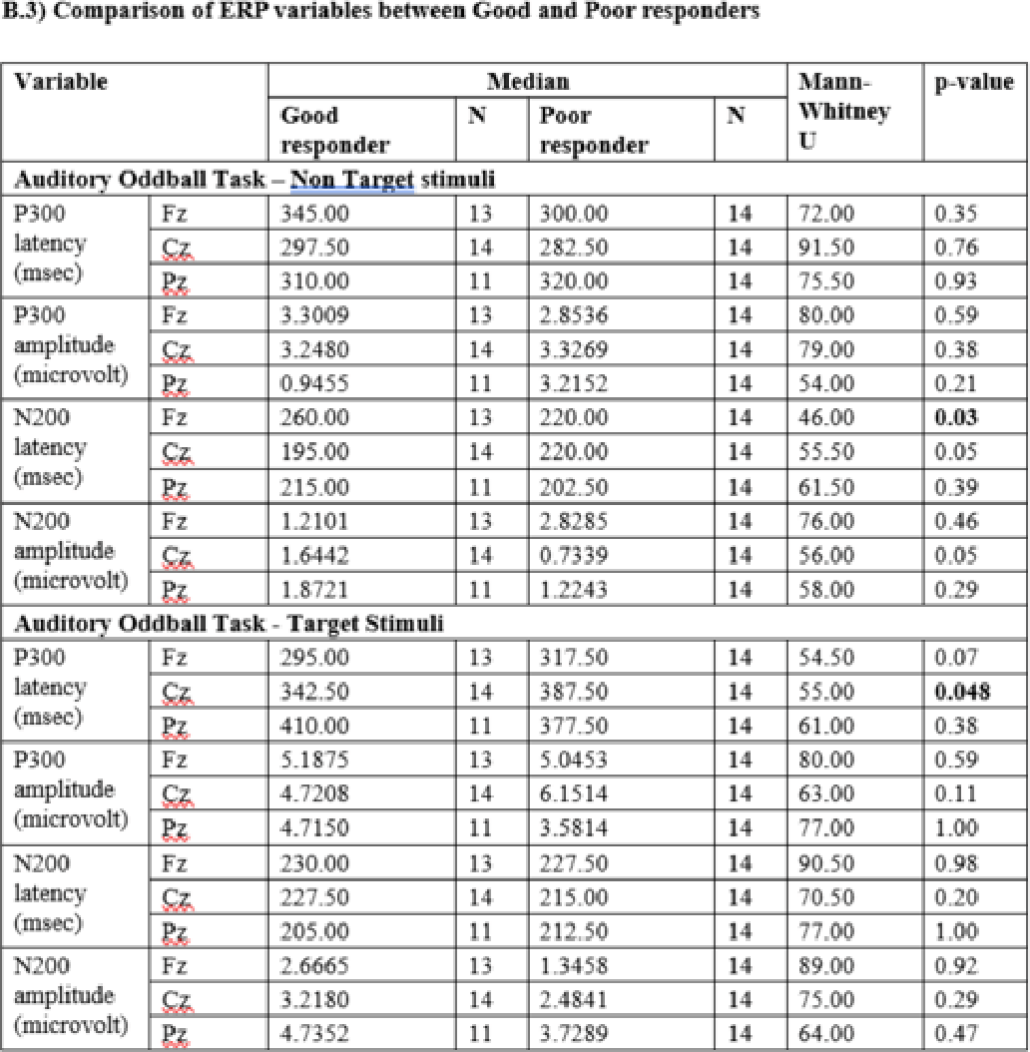
Image 2: Long description.

**Conclusions:**

There are differences in Evoked response potentials in Cz and Pz channels between good and poor responders. This underscores the importance of further research on neurophysiological markers as a predictive biomarker to various treatment modalities for ADHD.

**Disclosure of Interest:**

None Declared

## Long descriptions

### Image 1: Long description

The horizontal X-axis is labeled Time in m s e c, ranging from negative 1000 to approximately 2000, with a vertical zero line marking the stimulus onset. The vertical Y-axis is labeled Potential in mu V, ranging from negative 8 to positive 8.

* From negative 1000 to 0 m s e c, the blue waveform shows high-frequency oscillations between negative 5 and positive 3 mu V.

* At 0 m s e c, the waveform is near 0 mu V.

* Between 0 and 500 m s e c, there is a sharp positive peak reaching approximately 5 mu V followed by a return toward the baseline.

* From 500 to 1995 m s e c, the signal continues to oscillate with varying amplitudes, generally staying between negative 4 and positive 4 mu V.

* A small label C z is positioned in the lower-left quadrant near the negative 400 m s e c mark.

Navigate back to Image 1:.

### Image 2: Long description

The table is titled B.3 Comparison of E R P variables between Good and Poor responders.

Header Structure:

Columns from left to right are Variable, Median (subdivided into Good responder, N, Poor responder, N), Mann-Whitney U, and p-value.

Section 1: Auditory Oddball Task - Non Target stimuli

* P300 latency (msec) at Fz, Cz, Pz: Good responders range from 297.50 to 345.00; Poor responders range from 282.50 to 320.00. All p-values are non-significant (0.35 to 0.93).

* P300 amplitude (microvolt) at Fz, Cz, Pz: Good responders range from 0.9455 to 3.3009; Poor responders range from 2.8536 to 3.3269. All p-values are non-significant.

* N200 latency (msec) at Fz, Cz, Pz: Significant difference at Fz (Good: 260.00, Poor: 220.00, p-value: 0.03) and borderline at Cz (p-value: 0.05).

* N200 amplitude (microvolt) at Fz, Cz, Pz: Borderline significance at Cz (p-value: 0.05).

Section 2: Auditory Oddball Task - Target Stimuli

* P300 latency (msec) at Fz, Cz, Pz: Significant difference at Cz (Good: 342.50, Poor: 387.50, p-value: 0.048).

* P300 amplitude (microvolt) at Fz, Cz, Pz: Values range from 3.5814 to 6.1514 across groups; no significant p-values.

* N200 latency (msec) at Fz, Cz, Pz: No significant differences; p-values range from 0.20 to 1.00.

* N200 amplitude (microvolt) at Fz, Cz, Pz: No significant differences; p-values range from 0.29 to 0.92.

Sample sizes (N) are consistently 11 to 14 for Good responders and 14 for Poor responders.

Navigate back to Image 2:.

